# Radiological Anatomy of the Pelvis and Pelvic Limb of the Greater Cane Rat (*Thryonomys swinderianus*)

**DOI:** 10.1155/2024/5998717

**Published:** 2024-02-21

**Authors:** Faraja H. Mpagike, Modesta Makungu

**Affiliations:** Department of Veterinary Surgery and Theriogenology, Sokoine University of Agriculture, P.O. Box 3020, Chuo Kikuu, Morogoro, Tanzania

## Abstract

Greater cane rats (*Thryonomys swinderianus*) are now being captive reared and domesticated in the sub-Saharan Africa because of increase in their demand for biomedical research and traditional medicine and as a source of meat. This research was performed to provide the normal radiological anatomy of the pelvis and pelvic limb in greater cane rats for a reference in biomedical research, in anatomical studies, and in clinical use. Radiological examination of the pelvis and right pelvic limb was done in five greater cane rats. Radiological results were correlated with bones of the pelvis and right pelvic limb. The pelvic bone had a prominent caudoventral iliac spine. The pelvic symphysis was long and obturator foramina appeared teardrop-shaped elongated craniocaudally. The prominent major trochanter extended proximally higher than the femoral head. The middle third of the body of the tibia presented a very prominent tuberosity. In all specimens, the tarsal sesamoid bone was visualised. Mineralised popliteal sesamoid bone was not visualised. In male greater cane rats, the os penis was visualised. The lateral and medial menisci of the stifle joint were seen with ossicles. The first metatarsal bone was rudimentary with greater plantar divergence. Despite of the pelvic limb and pelvis of greater cane rats sharing anatomical structures with other rodents, it retains some exceptional anatomical features. Findings of this study will serve as a reference for anatomical studies, clinical veterinary practice, and in biomedical research.

## 1. Introduction

Greater cane rats (*Thryonomys swinderianus*) are mostly found in the southern part of the Saharan desert around streams, rivers, and irrigation dams and at the edges of marshes and wetlands [1–4]. Their distribution is primarily determined by the occurrence of thick cane-like grasses [2–4]. Greater cane rats are herbivores and feed mainly on roots and stems of grasses [1, 5]. Furthermore, in the wild, they feed on fruits, nuts, and seeds [6]. In captivity, greater cane rats are usually fed with forage and supplemented with tubers, underground stems, grains, and fruits [2]. They are nocturnal animals being observed to burrow underground or make nests from grasses [7]. Furthermore, they can run very fast [8] and are great divers and swimmers [7]. The average body weight of females and males greater cane rats have been reported to be 3.5 kg and 4.5 kg, respectively [7]. However, males have been documented to weigh up to 9 kg [7].

Greater cane rats are now being captive reared and domesticated in the sub-Saharan Africa [1, 3, 9, 10] because of increase in their demand for biomedical research [11] and traditional medicine [8, 12] and as a source of meat [9, 10]. The meat from cane rats is very delicious with high nutritional value and widely accepted [3]. Furthermore, it is high in protein and mineral content and very low in cholesterol [2]. Parts of the body of the cane rat such as the pancreas and hairs have been reported to be utilised in traditional medicine in the management of wounds, diabetes, and infertility in women [8, 12]. Their small size accelerates their use in biomedical research [11].

Several authors have reported musculoskeletal diseases and conditions in cane rats such as soft tissue wounds, fractures, and neoplasia like osteosarcoma, which threatens their survival [13–15]. Radiography is the diagnostic imaging modality, which is commonly used in routine health checkups and for monitoring, detection, and evaluation of musculoskeletal diseases in captive animals [16]. In cane rats, radiography is performed infrequently, which is partly being contributed by the lack of description of their normal radiological anatomy. Understanding the normal radiological anatomy of specific species and high standard radiographs are significant for accurate radiological diagnosis [16]. Normal radiological anatomy of the pelvis and pelvic limb in other rodents [17–20] and captive animals such as the hedgehog [21], red panda [22], coati [23], ring-tailed lemur [24], and nondomestic cats [25] have been reported, which serve as a reference for identification of musculoskeletal conditions and diseases.

There is a scarce literature on the gross osteology of the pelvis and pelvic limb of cane rats [26]. Furthermore, to the greatest of our knowledge, the report on the normal radiological anatomy of the pelvis and pelvic limb is not available for the cane rat. This study was performed with the aim of describing the normal radiological anatomy of the pelvis and pelvic limb in greater cane rats so as to provide a reference for anatomical studies, clinical veterinary practice, and in biomedical research.

## 2. Materials and Methods

### 2.1. Greater Cane Rats

Three male and two female greater cane rats (*Thryonomys swinderianus*) of a maximum weight of 7.0 kg and a minimum weight of 4.3 kg (mean: 5.7 ± 1.3 kg) were imaged. Animals were dead without a sign of musculoskeletal disease involving the pelvis and right pelvic limb on macroscopic and radiologic examinations. The death of cane rats was not associated with this research.

### 2.2. Radiography

The Roller 30 X-ray equipment (Smam X-ray Equipments, Italy) was used in this study. Nongrid exposure factors of 46 kVp to 48 kVp and mAs of 2.5 were used to image the pelvis and right pelvic limb. ColentaHighCapXr® (Fujifilm Corporation, Japan), computed radiography (CR) system, was used to capture images. For the pelvis, in all specimens, a ventrodorsal (VD) radiographic view was acquired with cane rats in dorsal recumbency. For the right pelvic limb, a mediolateral (ML) radiographic view was obtained with the cane rat in a right lateral recumbency, whereas a caudocranial (CdCr) radiographic view was taken with the cane rat in a sternal recumbency. Furthermore, a proximodistal radiographic view of the medial and lateral menisci of the right stifle joints was taken.

After radiologic examination, bones of the right pelvic limb and pelvis were prepared from cadavers and correlated with the radiological findings. CANON PC1192® digital camera (Canon Inc., Tokyo, Japan) was used to capture photographs of each bone. Nomina Anatomia Veterinaria was used for nomenclature [27], and earlier reports in domestic animals [28, 29] were used to identify functions, origins, and insertions of the pelvis and pelvic limb muscles. Approval of this research was granted by the Research, Innovation, and Publication Committee of the College of Veterinary Medicine and Biomedical Sciences of the Sokoine University of Agriculture.

## 3. Results

### 3.1. Pelvis

**Figure 1:**
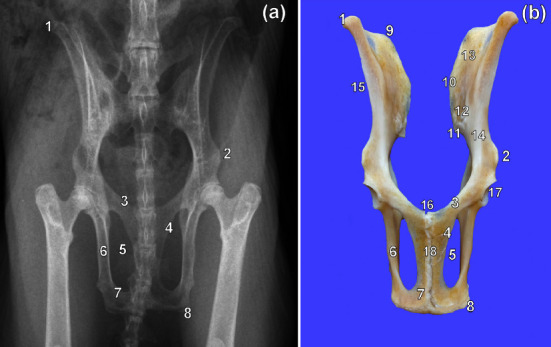
Ventrodorsal radiographic view (a) and ventral photographic view (b) of the pelvis of a greater cane rat. 1. Cranial ventral iliac spine; 2. caudal ventral iliac spine; 3. cranial ramus of the pubic bone; 4. caudal ramus of the pubic bone; 5. obturator foramen; 6. body of Ischium; 7. ischiatic table; 8. ischiatic tuberosity; 9. iliac crest; 10. cranial dorsal iliac spine; 11. caudal dorsal iliac spine; 12. auricular surface; 13. iliac surface; 14. body of ilium; 15. wing of ilium; 16. pecten of pubic bone; 17. acetabulum; 18. pelvic symphysis.

Caudal ventral iliac spine was conspicuous and the ilial wings were wide on the VD view (Figure 1). The ilia articulated with the first sacral vertebra (Figure 1(a)). The cranial opening of the pelvis, *Apertura pelvis cranialis*, was rounded (Figure 1(a)) and the pelvic symphysis was long (Figure 1). The obturator foramina appeared teardrop-shaped elongated craniocaudally (Figure 1). The ischial body was flattened mediolaterally, straight, relatively long, and parallel with one another (Figure 1). Ischiatic tuberosities were less prominent (Figure 1). The caudal and cranial rami of the pubic bone were broad (Figure 1). The acetabula were C-shaped (Figure 1).

### 3.2. Femur

**Figure 2:**
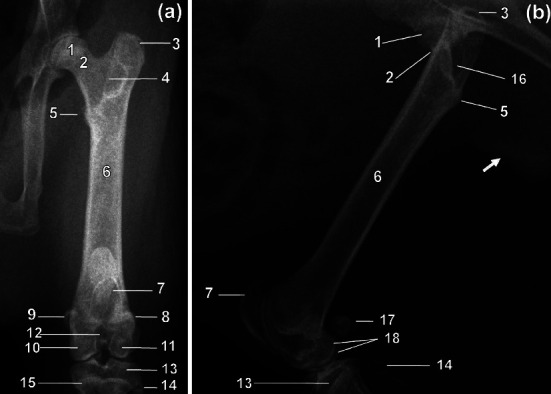
Caudocranial (a) and mediolateral (b) radiographic views of the right femur of a greater cane rat. 1. Head; 2. neck; 3. major trochanter; 4. intertrochanteric crest; 5. minor trochanter; 6. diaphysis; 7. patella; 8. lateral sesamoid bone of the gastrocnemius muscle; 9. medial sesamoid bone of the gastrocnemius muscle; 10. medial condyle; 11. lateral condyle; 12. intercondylar fossa; 13. tibia; 14. fibula; 15. proximal physis of the tibia; 16. trochanteric fossa; 17. superimposed lateral and medial sesamoid bones of the gastrocnemius muscle (lateral and medial fabellae); 18. superimposed lateral and medial condyles. Os penis (white arrow).

On the CdCr and ML views, the femur was fairly straight and slender (Figure 2). The diaphyseal cortices were almost of the same width (Figure 2). The medially directed femoral head was more or less rounded (Figure 2). The femoral neck was distinct and relatively long (Figure 2). The major trochanter was prominent and extended proximally higher than the femoral head (Figure 2). The caudomedially positioned minor trochanter was less prominent (Figure 2). Intertrochanteric crest connected minor and major trochanters (Figure 2). The former was visualised as a thin line of bone opacity slanting from proximolateral to distomedial on the CdCr view (Figure 2(a)). The third trochanter was not visualised (Figure 2).

### 3.3. Tibia and Fibula

**Figure 3:**
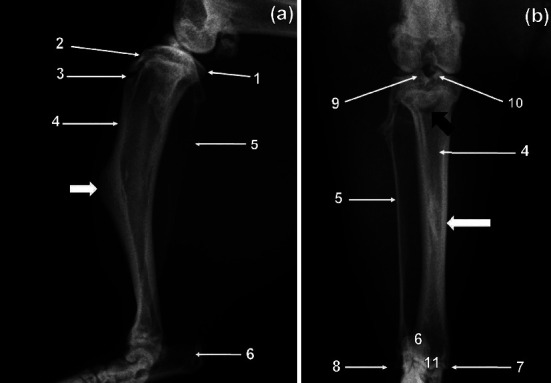
Mediolateral (a) and caudocranial (b) radiographic views of the right tibia and fibula of a greater cane rat. 1. Medial condyle; 2. tibial tuberosity; 3. physis of the tibial tuberosity; 4. tibia; 5. fibula; 6. calcaneous; 7. medial malleolus of the tibia; 8. lateral malleolus of the fibula; 9. lateral intercondylar tubercle; 10. medial intercondylar tubercle; 11. cochlea of the tibia. The prominent tuberosity is shown by a short white arrow. A crescent-shaped radiolucent area representing the physis of the tibial tuberosity is indicated by a black arrow. An increased area of bone opacity is indicated by a long white arrow.

The two bones were unfused in all specimens (Figure 3). The tibia was larger compared to fibula (Figure 3). The former was wider craniocaudally and flattened mediolaterally (Figure 3). The condyles of the tibia were wider than the cochlea of the tibia (Figure 3(b)). The fibula was wider craniocaudally and narrower mediolaterally (Figure 3). The craniocaudal width of the bone decreased from proximal to distal (Figure 3). The tibial tuberosity was less conspicuous, whereas the intercondylar eminence remained conspicuous on the ML view (Figure 3(a)). The tibia bent cranially on its longitudinal axis (Figure 3(a)) and presented a very prominent tuberosity (Figure 3(a)) on its cranial surface at the middle third of the body. Cranial cortex of the tibia was thicker than the caudal cortex (Figure 3(a)). The fibula was wide with its proximal and distal extremities superimposed on the proximal and distal extremities, respectively, of the tibia (Figure 3(a)). On the CdCr view, the tibia appeared fairly straight (Figure 3(b)). The intercondylar tubercles were prominent and of almost of the same height (Figure 3(b)). Proximal extremity of the tibia presented a crescent-shaped radiolucent area which corresponded to the physis of the tibial tuberosity (Figure 3(b)). Increased area of bone opacity was visualised in the middle third of the body of the tibia (Figure 3(b)) as a result of the presence of the very prominent tuberosity (Figure 3(a)). The lateral malleolus of the fibula appeared comma shaped whereas the medial malleolus of the tibia was short and directed distally (Figure 3(b)).

### 3.4. Tarsus

**Figure 4:**
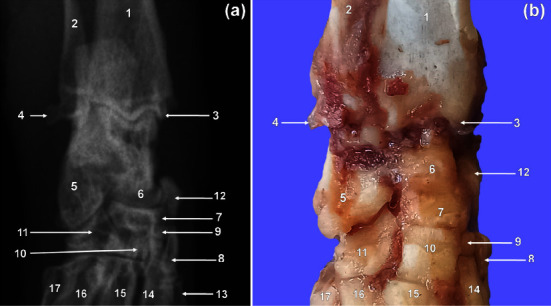
Plantarodorsal radiographic view of the right tarsus of a greater cane rat (a). Dorsal photographic view of the right tarsus of a greater cane rat (b). 1. Tibia; 2. fibula; 3. medial malleolus of the tibia; 4. lateral malleolus of the fibula; 5. calcaneus; 6. talus; 7. central tarsal bone; 8. TI; 9. TII; 10. TIII; 11. TIV; 12. tarsal sesamoid bone; 13. MTI; 14. MTII; 15. MTIII; 16. MTIV; 17. MTV.

**Figure 5:**
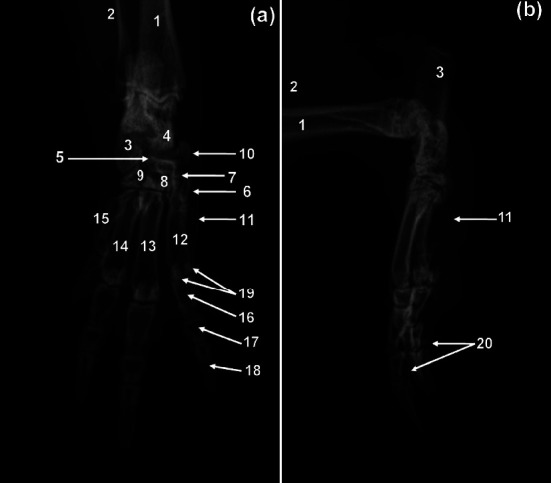
Plantarodorsal (a) and mediolateral (b) radiographic views of the right pes of a greater cane rat. 1. Tibia; 2. fibula; 3. calcaneous; 4. talus; 5. central; 6. TI; 7. TII; 8. TIII; 9. TIV; 10. tarsal sesamoid bone; 11. MTI; 12. MTII; 13. MTIII; 14. MTIV; 15. MTV; 16. PI; 17. PII; 18. PIII; 19. axial and abaxial proximal sesamoid bones; 20. distal sesamoid bones.

Seven bones were visualised and organised in three rows specifically; the proximal, middle, and distal rows (Figure 4). The calcaneus and talus bones were included in the proximal row (Figure 4). The largest bone, calcaneus, was situated plantarly and laterally to the talus (Figures 4 and 5). The latter stood the second in size (Figure 4). The middle row contained the third largest bone, the central (Figure 4). The distal row (Figure 4) consisted of tarsal (T) bones I–IV. Distally, the central bone had three more or less flat articular facets for articulation with the TII–IV (Figure 4). Medially, the central articulated with the tarsal sesamoid bone (Figure 4). Plantarly, it presented a boat-shaped tuberosity lengthened proximodistally. The TIV was the largest, whereas the TII was the smallest in the distal row. The furthermost medial bone, TI, was the third largest (Figure 4).

### 3.5. Metatarsal Bones and Digits

Metatarsal (MT) bones I–V were visualised. The MTI was rudimentary without phalanges whereas MTII–V were fully developed with widely spread digits. The former had a greater plantar deviation from MTII–V. The MTV was directed mediolaterally. Each of the digits II–V presented three phalanges, namely, the proximal (PI), middle (PII), and distal (PIII) phalanges (Figure 5(a)). The MTI was visualised superimposed on the MTII on the PlD view (Figure 5(a)).

### 3.6. Sesamoid Bones

**Figure 6:**
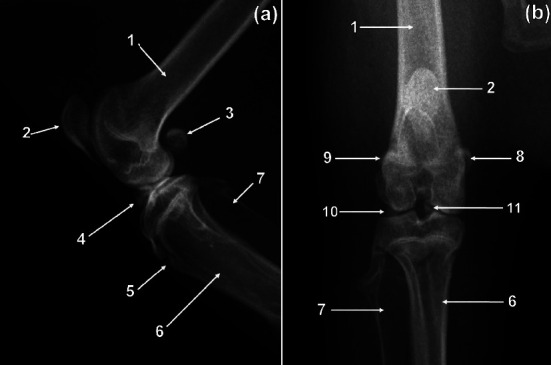
Mediolateral (a) and caudocranial (b) radiographic views of the right stifle of a greater cane rat. 1. Femur; 2. patella; 3. lateral and medial fabellae; 4. superimposed lateral and medial meniscal ossicles; 5. physis of the tibial tuberosity; 6. tibia; 7. fibula; 8. medial fabella; 9. lateral fabella; 10. lateral meniscal ossicle; 11. medial meniscal ossicle.

The patella had a sharp and extended apex (Figure 6). The former had a tuberosity on the cranial surface (Figure 6(a)). The tuberosity of the patella was seen as an area of increased bone opacity on the ML view (Figure 6(a)). The patella was poorly visualised on the CdCr view of the stifle as a result of its superimposition on the distal third of the body of the femur (Figure 6(b)). A tarsal sesamoid bone was visualised in all cane rats (Figures 4 and 5). The tarsal sesamoid bone appeared comma shaped on the PlD view (Figure 4). Lateral and medial sesamoid bones of the gastrocnemius muscle (lateral and medial fabellae) were seen in all animals (Figure 6). The two fabellae were visualised superimposed on the ML view (Figure 6(a)). Each metatarsophalangeal joint of digits II–V had paired proximal sesamoid bones (Figure 5(a)). In addition, each distal interphalangeal joint of digits II–V had a distal sesamoid bone (Figure 5(b)). The mineralized sesamoid bone for the popliteal muscle was not seen (Figure 6).

### 3.7. Other Findings

**Figure 7:**
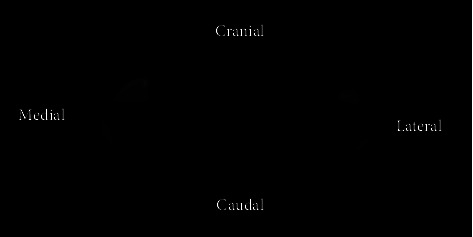
Proximodistal radiographic view of the medial and lateral menisci of the right stifle of a greater cane rat. Note the location of the ossicles in the cranial horn of menisci. Note also the triangular and ovoid-shaped medial and lateral menisci, respectively.

In male greater cane rats, the os penis was visualised on the ML view of the femur (Figure 2(b)). The bone was visualised as a fusiform area of bone opacity elongated craniocaudally (Figure 2(b)). Ossicles were observed in the medial and lateral menisci of the stifle joint in all specimens (Figure 7). The former were located in the cranial horn of the medial and lateral menisci (Figure 7). On the proximodistal view, the medial meniscal ossicle appeared triangular shaped with a trabecular pattern (Figure 7), whereas the lateral meniscal ossicle appeared ovoid shaped with a trabecular pattern (Figure 7). The medial meniscal ossicle was larger than the lateral meniscal ossicle (Figure 7). The lateral and medial meniscal ossicles were superimposed and appeared as a triangular area of bone opacity in the cranial part of the joint on the ML view (Figure 6(a)). The lateral and medial meniscal ossicles were poorly visualized due to their superimposition on the femur and tibia on the CdCr view of the stifle (Figure 6(b)).

## 4. Discussion

Greater cane rats have been observed to run very fast [8] and are capable of jumping [30]. Furthermore, they are great divers and swimmers [7, 8] and have been documented to sit upright during feeding [8]. Although greater cane rats are fossorial animals, they do not usually dig their own burrows [8]. They have been observed to hide in holes made by other wild animals [8]. Cane rats normally scrape small depressions that are saucer shaped between vegetation [8].

The dorsolateral oriented gluteal surface seen in greater cane rats is related to postural habit of sitting and squatting [31]. Cane rats have been documented to sit upright during feeding [8]. During sitting the craniomedially and dorsolaterally directed iliacus and glutei muscles, respectively, are tensed to prevent falling caudally and cranially, respectively [31]. The visualization of less prominent ischial tuberosities in greater cane rats is more or less analogous to lowland pacas [18, 20, 32] and capybaras [19]. Less prominent ischial tuberosities have been reported to be associated with jumping and climbing [33]. Both the lowland paca, capybara, and greater cane rat are capable of jumping. The lowland paca ranges in weight from 6 kg to 12 kg and is considered a terrestrial rodent though it is a good swimmer [32]. The capybara is a wild semiaquatic largest rodent in the world weighing between 30 kg and 100 kg [19, 32]. However, long runs in capybara have been reported to initiate hyperthermia and fatigue [19]. Moreover, the teardrop-shaped obturator foramina elongated craniocaudally in greater cane rats are similarly to capybaras [19] and lowland pacas [18, 20]. The elongated obturator foramina are the result of the presence of long ischia and caudal rami of pubic bone. The relatively long pelvis symphysis in greater cane rats is comparable to that which is being observed in lowland pacas and capybaras [19, 20] and is associated with adaptation for power in aquatic locomotion [34]. In a study which involved three species of African mole-rats, the ischial tuberosity was prominent, obturator foramen was rounded to ovoid, and pelvic symphysis was relatively short [35]. The prominent ischial tuberosity in African mole-rats most likely indicates an adaptation for digging as it was explained in a wild rabbit [36]. The African mole-rat is a highly specialised fossorial rodent that lives in its own tunnel system [37, 38].

The rounded appearance of the cranial opening of the pelvis on the VD view in greater cane rats is unlike the lowland paca [18, 20] and capybara [19]. In lowland pacas [18, 20] and capybaras [19], the cranial opening of the pelvis on the VD view appears ovoid in shape, indicating dolicopelvic animals [20]. The radiographic visualisation of broad ilial wings and well-marked major trochanters, which extended proximally higher than the femoral heads in greater cane rats, enhances the extensor function of the gluteus medius muscle [33] and is more or less similar to lowland pacas and capybaras [18–20, 32]. The proximal extension of the major trochanter than the head of the femur and the broad ilial wings further reduce the moment of the gluteus medius muscle in abduction and medial rotation of the femur, which is a feature in cursorial animals [33, 39]. The ilial wing and major trochanter provide the origin and insertion, respectively, of the gluteus medius muscle [29]. The latter is the strong extensor of the hip joint [29]. In a study, which involved African mole-rats in all species, major trochanters did not extend proximally beyond the head of the femur [35]. Furthermore, the ilial wings appeared relatively narrow [35, 37, 38]. In African Viverridae (carnivora) the insertion of the gluteus medius muscle at the same level or distal to the head of the femur was observed to enhance the abduction function of the gluteus medius muscle and was a feature in burrowing species [33]. Abduction of the pelvic limbs permits the animal to sprout the earth backwards with its thoracic limbs between its pelvic limbs during burrowing [33]. Furthermore, it is important for burrowing activity involving the pelvic limbs [33]. The very conspicuous caudal ventral iliac spine observed in this species on the VD view is contrary to lowland pacas [20, 32] and capybaras [19]. The caudal ventral iliac spine offers the origin of the rectus femoris muscle, part of the quadriceps femoris muscle [29]. The former indicates the power of the rectus femoris muscle in extension of the stifle joint as an adaptation for jumping [24].

The radiological visualisation of a fairly straight and slender femur, with a rounded femoral head and distinct relatively long femoral neck in greater cane rats is more or less similar to the capybara [19] and lowland paca [18, 40]. In this study, the third trochanter was not visualised in all greater cane rats similar to a previous reported study in cane rats [26]. Furthermore, the absence of visualisation of the third trochanter in greater cane rats is analogous to a reported study in the capybara and lowland paca [32]. However, in other reported studies, which involved the lowland paca [18, 19, 40] and capybara [19], the third trochanter was seen as a very small projection. In a study that involved three species of African mole-rats, the third trochanter was prominent in all species [35]. Furthermore, the third trochanter was reported to be well developed in scratch diggers and chisel-tooth diggers African mole-rats [38]. The presence of a third trochanter in African mole-rats was related to fossorial adaptation [38].

The patella is the largest sesamoid bone interposed in the tendon of the quadriceps femoris muscle [29]. The pointed and elongated apex of the patella observed in this study on the ML and CdCr views of the stifle joint is comparable to the lowland paca [18, 32]. The tuberosity on the patella seen in this species shows the power of the quadriceps femoris muscle in extension of the stifle during jumping [24]. Radiological visualization of meniscal ossicles and involvement of the ossicle in the cranial horn of both the lateral and medial menisci in greater cane rats has also been reported in the lowland paca [18, 32], capybara [32], and other species of rodents [32]. Similar to greater cane rats, lowland pacas and capybaras have been reported to have two meniscal ossicles, one in the cranial horn of the medial meniscus and the other in the cranial horn of the lateral meniscus [32]. In other species of rodents such as the common rat, the lateral meniscus has been reported to have two ossicles, i.e., one ossicle is situated in the cranial horn and the other ossicle is located in the caudal horn [32]. The meniscal ossicle is related to jumping and running [41] and its normal finding in greater cane rats. The nonappearance of the sesamoid bone for the popliteal muscle in greater cane rats is similar to the capybara [19] and lowland paca [18]. The visualisation of both the lateral and medial fabellae in this species is like in common rats [32] but different from capybaras [19], lowland pacas [18], and African mole-rats [35]. The lateral and medial fabellae were not observed in a report, which involved three species of African mole-rats [35]. In a computed tomography (CT) study, both the medial and lateral fabellae were not visualized in the capybara, whereas the medial fabella was not visualized in the lowland paca [32]. The lack of visualization of mineralised sesamoid bones on radiological examination has been reported to be nonsignificant clinically [42].

The visualization of the prominent tuberosity on the cranial surface of the tibia in the middle third of the body in greater cane rats is contrary to lowland pacas [18] and capybaras [19] and relates with the power of the semitendinosus muscle in extension of the pelvic limb as an adaptation for jumping. The tuberosity offers the insertion of the semitendinosus muscle that extends the tarsal, stifle, and hip joints when the pelvic limb is bearing weight [29]. When the pelvic limb is not bearing weight, the semitendinosus muscle flexes the stifle joint [29]. The tuberosity has also been observed in African mole-rats [35, 38, 43], however, is not as prominent as in greater cane rats. In African mole-rats, the tuberosity most likely is related with fossorial adaptation. Studies on pelvic limb morphology of the African mole-rat [35, 37, 38, 43] indicated the fusion of the fibula and tibia around the midshaft in majority of species and was associated with fossoriality. The fusion of the tibia and fibula in African mole-rats increases bone resistance to torsional loads and bending during burrowing [38, 44]. In all specimens of greater cane rats, the fusion of the fibula and tibia was not observed, which is analogous to reported studies in the lowland paca [18, 40], capybara [19], and naked mole-rat [38]. Lack of fusion of tibia and fibula permits an increased range of movement of the pelvic limb [38].

The radiological visualisation of seven tarsal bones and a tarsal sesamoid bone in greater cane rats is comparable to the capybara [19, 32] and lowland paca [40]. In this study, all greater cane rats had five MT bones similar to a reported study in the lowland paca [40], which is different from the capybara [19, 32]. The latter had only four MT bones [19, 32]. Moreover, the mediolaterally directed MTV in the greater cane rat is similar to the lowland paca [40]. The MTI was also reported to be reduced in the lowland paca [40]; however, it had proximal and distal phalanges [40], contrary to greater cane rats. The decrease in the digit number is related with either a cursorial or fossorial adaptations [44, 45]. The presence of relatively short and stout MT bones with rudimentary MTI in greater cane rats is most likely related to fossorial adaptation [44]. In cursorial animals, the MT bones are elongated and close to each other with the MTI being reduced or absent [46]. The radiological visualisation of the os penis in this species should not be mistaken as soft tissue mineralisation. The os penis is also seen in the domestic dog [47], ring-tailed lemur [24], red panda [22], and coati [23] on radiological examination.

## 5. Conclusions

The pelvis and pelvic limb morphology of greater cane rats showed adaptation to cursorial, aquatic, and fossorial habits. Furthermore, apart from sharing anatomical structures with other rodents, it retains some exceptional anatomical features. Findings of this study will serve as a reference for clinical use and biomedical research and in anatomical studies.

## Data Availability

The data used to support the findings of this study are available from the corresponding author upon reasonable request.
